# Prospective Memory Performance of Autistic Adults in Everyday Life: The Role of Stress and Motivation

**DOI:** 10.1002/aur.70057

**Published:** 2025-05-29

**Authors:** Larissa L. Faustmann, Mareike Altgassen

**Affiliations:** ^1^ Department of Psychology Johannes Gutenberg University Mainz Mainz Germany

**Keywords:** autism, everyday life, experience sampling method, high‐functioning autism, motivation, prospective memory, stress

## Abstract

Prospective memory (PM) is the ability to remember to carry out intended actions in the future. The present study investigated the PM performance of autistic adults in everyday life. A total of 29 autistic participants and 30 controls matched for age, gender, and cognitive abilities completed various PM tasks (time‐based vs. event‐based; externally‐assigned vs. self‐assigned), integrated into a three‐day Experience Sampling Method (ESM) assessment. The ESM survey assessed other activities performed during the 3 days, participants' motivation, daily routine, and perceived daily‐life stress. No group differences were found between autistic and control participants in any of the various PM tasks. Autistic participants did not differ from control participants in the types of everyday activities performed or in motivation, but showed higher levels of perceived stress. This is the first study to investigate PM performance of autistic individuals in everyday life. The results suggest that autistic adults show no PM difficulties in naturalistic PM tasks.

**Summary**

- Prospective memory (PM) is the ability to remember to carry out intended actions in the future. The present study investigated the prospective memory (PM) performance of autistic adults in everyday life.
- Twenty‐nine autistic and 30 control participants completed a series of PM tasks over a three‐day period. At the same time, they provided daily reports through questionnaires about other activities they performed, their motivations for these activities, and their experienced stress levels.
- Autistic participants did not differ from control participants in their PM performance. Both groups of participants engaged in similar types of daily activities. However, autistic individuals experienced higher levels of stress in their everyday lives compared to control participants.
- The results suggest that in contrast to some laboratory‐based tasks, autistic individuals do not experience difficulties with PM tasks in naturalistic settings.

## 1 Introduction

Autism spectrum disorders (ASD) are part of a group of neurodevelopmental conditions characterized by impairments in social interaction, communication, and a reduced behavioral flexibility that may become apparent in repetitive and restricted behavior and interests. The severity of symptoms differs in each individual and might change across the lifespan (APA 2013). Recent research suggests a 3:1 male‐to‐female ratio (Loomes et al. 2017; Wilson et al. 2016).

ASD are generally associated with executive dysfunction (Demetriou et al. 2018, see Hill 2004, for a review). Several studies have reported problems in cognitive flexibility in ASD (see Boyd et al. 2009; Geurts et al. 2009; Gioia et al. 2002; Mackinlay et al. 2006; but see Leung and Zakzanis 2014, for no impairments) and challenges in planning, such as organizing spaces or creating and adhering to a schedule (Happé et al. 2006; van den Bergh et al. 2014). However, it is not clear whether autistic individuals are impaired in planning or cognitive flexibility in general, or whether the observed difficulties may be attributed to possible moderators such as the severity of ASD symptoms, comorbid psychopathology, or general intelligence (Kenworthy et al. 2008; Olde Dubbelink and Geurts 2017).

Executive functions are highly relevant in mastering everyday life demands and in responding to novel or unexpected challenges. Accordingly, reduced executive functions might have a highly negative impact on the well‐being of autistic individuals. For example, a student with an ASD diagnosis may demonstrate excellent academic performance. Despite his achievements, without being able to maintain a schedule, it might be difficult for him to be successful in a less structured college setting without any help from teachers or his parents. Besides challenges with executive functioning, autistic individuals often experience difficulties with episodic memory, particularly when tasks require significant self‐initiated processing (see Griffin et al. 2022; Lind 2010).

Difficulties in meeting deadlines (e.g., Lambe et al. 2019) have been further related to challenges in prospective memory (PM). PM describes a cognitive ability that enables individuals to remember to execute an intended action at a certain point in the future. In research, a distinction is made between time‐based (i.e., remembering to perform an intended action at a certain point in time, for example, taking medication at 8:00 AM every day) and event‐based PM (i.e., remembering to perform an intended action when a specific cue is presented, for example, packing sports equipment when leaving the house for a soccer game; Einstein and McDaniel 1990).

PM comprises multiple processes and four phases (Kliegel et al. 2002): First, the individual has to plan which actions he/she wants to perform at a certain moment. This intention formation phase is mainly based on planning abilities. In the second phase, intention retention, the intention has to be kept in mind while the individual is engaged in other ongoing activities. The third phase of intention initiation requires monitoring to detect the cue indicating the appropriate moment to inhibit the ongoing activity and switch to the intended action. In the last (fourth) phase, intention execution, the planned action is finally carried out.

Given that executive functions and episodic memory are essential for successful PM performance (Einstein and McDaniel 1990; Martin et al. 2003), difficulties in PM in autistic individuals have been suggested. While several studies (Altgassen et al. 2009, 2019; Brandimonte et al. 2011; Henry et al. 2014; Kretschmer‐Trendowicz et al. 2014; Landsiedel and Williams 2020; Williams et al. 2013; Williams et al. 2014; Yi et al. 2014) indicate PM deficits in ASD, there is also evidence for intact PM (Altgassen et al. 2010; Altgassen and Koch 2014; Groenman et al. 2024; Henry et al. 2014; Sheppard et al. 2016; Williams et al. 2013). Indeed, PM difficulties seem to be mainly evident in time‐based tasks (children and adolescents: Altgassen et al. 2009, 2019; Williams et al. 2013; Henry et al. 2014; adults: Kretschmer‐Trendowicz et al. 2014; Williams et al. 2014; Landsiedel and Williams 2020), while mixed findings were found for event‐based PM performance (difficulties in PM: Brandimonte et al. 2011; Kretschmer‐Trendowicz et al. 2014; Yi et al. 2014 intact performance: Altgassen et al. 2010; Altgassen and Koch 2014; Henry et al. 2014; Sheppard et al. 2016; Williams et al. 2013). Time‐based PM tasks are generally assumed to put higher demands on executive control resources than event‐based tasks, as they do not include an external cue which may prompt automatic retrieval of the intention, but instead require the individual to keep track of the elapsing time (cf. Einstein and McDaniel 1996).

Past studies have examined PM in ASD almost exclusively in the laboratory, which may limit the applicability of their findings to everyday PM abilities. In fact, it has been observed in other populations with difficulties in executive functioning and episodic memory (e.g., older adults) that deficits in PM found in the laboratory do not necessarily emerge in naturalistic tasks that have to be completed in everyday life (e.g., remembering to post a letter or to call the experimenter at a certain time). With regards to typically developing younger and older adults' PM performance across settings, these seemingly paradoxical findings have been coined as age‐ PM paradox (Rendell and Craik 2000) and refer to the pattern that older adults perform inferior to younger adults in laboratory PM tasks, but perform comparably or even better in everyday settings (see Henry et al. 2004; Uttl 2008). More specifically, age benefits in everyday settings are typically observed for naturalistic experimenter‐assigned time‐based tasks (= PM tasks assigned by an experimenter that participants are required to carry out in an everyday life setting at a specific time), while no age effects have been found for event‐based naturalistic PM tasks and for participants own real‐life self‐assigned time‐based tasks (= PM tasks that are assigned by the participant themself and represent participant's own real‐life intentions that are carried out in their everyday lives; Schnitzspahn et al. 2020). It has been suggested that cognitive demands and motivational influences may differ across settings. For example, working memory seems to play a larger role in laboratory settings as compared to everyday settings. In contrast, perceived intention importance, the use of external memory aids, the length of the delay interval, and individuals' conscientiousness seem to be more relevant in everyday life (Rummel et al. 2023).

Recently, Groenman et al. (2024) integrated naturalistic experimenter‐assigned PM tasks into their laboratory‐based PM study (i.e., Amsterdam Breakfast Task). For the event‐based PM task, participants had to insert tokens into a basket after every computer task, while time‐based PM tasks involved participants reporting if they needed a break after an hour of testing. They found no differences between autistic and non‐autistic participants, neither in the laboratory nor the naturalistic PM tasks.

Laboratory PM tasks differ from PM tasks in naturalistic, everyday settings in their design (e.g., the complete absence of an experimenter‐set ongoing task or lack of control over ongoing tasks and other influencing factors by the experimenter) and, regarding self‐assigned PM tasks, also in how autonomously the participants can perform the tasks. In contrast to experimenter‐assigned PM tasks, self‐assigned tasks allow participants to a certain degree to choose which activity to perform and when to execute it. Self‐determination plays an important role in the development of intrinsic motivation (self‐determination theory; Deci and Ryan 1985).

So far, no study has investigated PM in autism outside the laboratory. To close this gap, the present study aims to examine autistic individuals' PM performance in everyday life using both naturalistic, experimenter‐assigned tasks as well as participants' own real‐life intentions (self‐assigned PM tasks). Investigating the latter may be particularly relevant in understanding the role of motivation on PM performance in ASD. Motivation might play a crucial role in how well individuals remember future tasks and commitments. High motivation may lead to increased attention (Engelmann and Pessoa 2014), better intention‐encoding strategies (Penningroth and Scott 2007) and might facilitate spontaneous intention retrieval triggered by the PM cue (Einstein et al. 2005), ultimately resulting in improved performance in PM.

Importantly, different self‐assigned PM tasks may vary with regard to their voluntariness and personal significance. An obligation is defined as an act or course of action to which a person is morally or legally bound (see Oxford University Press 2024), while leisure activities are generally viewed as enjoyable and freely chosen (e.g., Henderson 1984; Newman et al. 2014). Indeed, the lack of obligation has been repeatedly identified as a defining characteristic of leisure (Watkins and Bond 2007). Ryan and Deci (2002) suggested that leisure activities and obligations might be associated with different types of motivational processes (enjoyment‐based vs. obligation‐based intrinsic motivation).

Regarding motivational effects on PM in autism, Landsiedel and Williams (2020) showed that emphasizing the importance of the PM task over the ongoing task increased time‐based PM performance in autistic participants (but see Altgassen et al. (2019), for no effects of importance manipulations). Since self‐assigned PM tasks, compared to experimenter‐assigned tasks, are determined by the participants themselves and therefore are more likely to be intrinsically motivated (Deci and Ryan 1985; Ryan and Deci 2000), they may also be associated with greater personal significance which might improve the PM performance of autistic individuals in such tasks.

In contrast, experimenter‐assigned PM tasks, which involve a commitment to meeting deadlines, could be associated with increased stress for autistic individuals, who often rigidly adhere to routines (APA 2013) and may react sensitively to changes in their plans caused by external demands such as the experimenter‐assigned PM tasks. The same might apply to self‐assigned obligations, which could be experienced as more stressful for autistic individuals, as they may be perceived as limiting their degree of autonomy and may require adaptation to external demands and thereby a deviation from one's own plans and routines (e.g., Didden et al. 2008; Stoppelbein et al. 2016).

Stress is defined as a state of disturbed homeostasis that induces somatic and mental adaptive reactions, called “stress response” (McEwen 2007). Regarding ASD, impairments in cognitive flexibility and the ability to shift between tasks can also limit individuals' ability to cope with daily life demands and thereby exacerbate the impact of stressors on health (Kerns et al. 2015). Indeed, autistic individuals experience more lifetime stressors and generally perceive stressors as being more severe than typically developing adults (Bishop‐Fitzpatrick et al. 2015, 2017; Hirvikoski and Blomqvist 2015; McGillivray and Evert 2018; Moseley et al. 2021). Greater perceived stressor severity in ASD has also been related to poorer physical and mental health, experienced loneliness, and lower social support (Moseley et al. 2021). In addition, autistic people are less likely to seek social support or help (Hirvikoski and Blomqvist 2015), leading to chronically high stress levels that, in turn, may have a negative impact on physical and mental health (Slavich 2016).

Research has shown that chronic stress (Mika et al. 2012) might have a negative impact on executive functions, which are thought to play a crucial role in PM. Indeed, chronic stress seems to reduce PM performance in laboratory tasks (Chen et al. 2019; Eskildsen et al. 2015). Similarly, Ihle et al. (2012) demonstrated that better performance in an everyday PM task was associated with lower stress levels. In contrast, Stewart and McFarland (2020) found stronger chronic stress to be linked to improved monitoring behavior as well as better time‐based PM.

To date, there is no evidence on the relationship between stress and PM in the daily lives of autistic individuals. A highly effective method for evaluating how individuals respond to context‐specific daily stress is the Experience Sampling Method (ESM; Csikszentmihalyi and Larson 1987; also known as Ecological Momentary Assessment, EMA; Myin‐Germeys et al. 2018; Ambulatory Assessment, AA; Trull and Ebner‐Priemer 2013). ESM uses short questionnaires about momentary experiences which are presented to participants at various moments throughout the day. Diary methods for the assessment of self‐reported behaviors and experiences have gained importance in recent years, as they reduce memory biases and increase the ecological validity of reported behavior (Shiffman et al. 2008). ESM is typically used to assess subjective experiences of stress (Vaessen et al. 2023), motivation (e.g., Koudela‐Hamila et al. 2019), mood (Hegarty et al. 2016; Mueller et al. 2019), social behavior (Runyan et al. 2019), physical activity (e.g., Guérin et al. 2013) or psychiatric symptoms (Myin‐Germeys et al. 2018).

Despite the many benefits of ESM studies, only a few studies examined ASD (e.g., Chen et al. 2014; Feller et al. 2023; Kovac et al. 2016) and perceived stress in these individuals (e.g., Ilen et al. 2023; van der Linden et al. 2020; van der Linden et al. 2021; van der Oosterhout et al. 2021). It was shown that the association between unpleasant daily events and negative affect was more pronounced in adults with ASD compared to controls, indicating an elevated affective reactivity to stress related to events and activities in ASD (van der Linden et al. 2021). Ilen et al. (2023) demonstrated in a recent study that young autistic adults exhibited higher perceived stress levels with regard to their daily social context and activities compared to non‐autistic adults. Autistic individuals further reported using less adaptive and more maladaptive emotion regulation strategies. Hence, there seems to be an increased stress sensitivity related to everyday activities and insufficient emotion regulation strategies in autistic individuals. However, it remains unclear whether other characteristics of activities (such as their importance or plannedness) and individuals' general ability to implement planned activities (PM) affect the acute stress experience of individuals with ASD.

The present study set out to investigate PM performance in everyday life. Integrated into a three‐day ESM assessment, adults with and without ASD were asked to perform various time‐based and event‐based PM tasks in their daily lives. Some PM tasks were externally assigned by the experimenter; others were self‐assigned by the participants. Participants were requested to carry out two externally assigned PM tasks per day. One of the tasks was event‐based; the other one was time‐based (writing text‐messages in response to a target cue or time). In addition, participants were asked to define six own time‐based PM tasks (hence, six own self‐assigned, real‐life intentions) that they intended to perform across the next 3 days. At the end of the third day, they had to report if they executed the tasks as planned. When forming their intentions, participants were asked to state three intentions that could be classified as leisure activities, while the other three had to be considered personal obligations. Ryan and Deci (2002) suggested that leisure activities and obligations might be associated with different types of intrinsic motivation (enjoyment‐based vs. obligation‐based intrinsic motivation).

Taken together, several laboratory studies on PM have reported impairments in ASD (e.g., Altgassen et al. 2009, 2019; Henry et al. 2014; Kretschmer‐Trendowicz et al. 2014; Landsiedel and Williams 2020; Williams et al. 2013, 2014). The only study that investigated PM using a more naturalistic task within a lab‐based setting found no differences in performance between autistic and control participants (Groenman et al. 2024). The PM age paradox demonstrates that there can be differences in PM performance in specific groups depending on whether the tasks are laboratory‐based or conducted in everyday life. However, given the expected high levels of stress of autistic individuals in daily life (Bishop‐Fitzpatrick et al. 2015, 2017; Hirvikoski and Blomqvist 2015; McGillivray and Evert 2018; Moseley et al. 2021) and the additional demands on executive control functions that PM tasks in everyday life may require, we predicted intention execution in all tasks (experimenter‐assigned and self‐assigned) to be lower for autistic participants as compared to control participants. Importantly, this study will exclusively assess PM performance in everyday life.

New insights into the PM performance of autistic individuals could emerge when motivational aspects are taken into account in the design of the PM tasks. Therefore, in our study, we aimed to investigate whether PM performance differs when the PM tasks are experimenter‐assigned compared to self‐assigned PM tasks. We hypothesized that participants in both groups might be more motivated to engage in self‐assigned activities than in experimenter‐assigned PM tasks, leading them to potentially execute the self‐assigned activities more successfully. Furthermore, we will explore potential differences in the execution of self‐assigned leisure activities versus obligations.

Through the ESM survey, we aim to gain additional insights into the daily structure of autistic individuals by capturing how often activities were planned in advance and whether these activities were carried out according to the planned schedule. Previous ESM studies (Ilen et al. 2023; van der Linden et al. 2021) have shown that certain everyday events (e.g., social gatherings) can lead to increased stress levels in autistic individuals. We aim to explore how motivation and importance of activities may affect stress perception in individuals with ASD. Furthermore, we aim to explore the relationship between perceived stress and PM performance in daily life. Chronic stress could potentially affect the PM performance of participants with ASD, as it may impair executive functioning (Chen et al. 2019; Stewart and McFarland 2020) and thereby reduce processes involved in PM such as working memory, planning, and switching abilities (Kliegel et al. 2002).

## 2 Method

### 2.1 Participants

In total, 29 autistic adults (age *M* = 33.62, SD = 13.06; 16 women, 11 men and two diverse individuals) and 30 non‐autistic adults (age *M* = 32.6, SD = 11.71; 19 women and 11 men) took part in the study. Groups were parallel for age, gender, and highest education degree. ASD participants were recruited by contacting mental healthcare facilities, self‐help groups in Germany, Austria, and German‐speaking Switzerland, and via social media (Facebook). Inclusion criteria were being aged between 18 and 69 years, German mother tongue, and availability of a smartphone with mobile internet access. All participants in the ASD group had formal diagnoses of the autism spectrum. Exclusion criteria were the presence of specific severe psychiatric disorders (such as an acute schizophrenic, manic or severe depressive episode or neurological illnesses and in the control group additionally the absence of a diagnosis of the autism spectrum).

Comorbid disorders in the ASD group were affective disorders such as major depression (40%), anxiety disorders (7%) and ADHD (10%). Diagnoses in the control group were major depression (7%) and anxiety disorders (3%).

Among the autistic participants, 17% reported having obtained a secondary school diploma or completed vocational training, 43% had earned a general or subject‐specific university entrance qualification, and 40% had completed a university degree.

Participant characteristics and group matching statistics are presented in Table 1. All participants gave written informed consent prior to taking part in the study. Participants received 7.50 Euro for taking part in the study. The study was conducted in line with the Helsinki declaration. Ethical approval for the study was obtained from the appropriate university ethics committee.

**TABLE 1:** Individual difference variables.

	ASD (*n* = 29)	CG (*n* = 30)	*F* (df)	*p*	*η* ^2^
	*M* (SD)	*M* (SD)			
Age	33.62 (13.06)	32.60 (11.71)	0.10 (1, 57)	0.753	0.002
AQ	24.17 (3.81)	8.87 (3.41)	264.08 (1, 57)	0.001	0.869
WAIS‐IV					
Verbal ability	12.4 (2.76)	11.45 (2.27)	1.25 (1, 57)	0.268	0.139
Non‐verbal ability	11.67 (3.02)	9.76 (2.31)	9.14 (1.57)	0.009	0.302

*Note:* For the WAIS subtests, results are reported in age normed scaled scores (*M* = 10, SD = 3; range 1–19).

Abbreviations: ASD, autism spectrum disorders; CG, control group.

### 2.2 Tests and Procedure

#### 2.2.1 Individual Difference Variables

The Autism Spectrum Quotient Test—Short Version, Freitag et al. 2007) is a screening questionnaire that assesses the subjective severity of ASD symptoms. It comprises 33 questions with a 4‐level Likertscale: Strongly Agree, Slightly Agree, Slightly Disagree, Strongly Disagree. A score of 17 or more indicates clinically significant levels of autistic traits.

To assess participants' verbal and non‐verbal abilities, the vocabulary and matrices reasoning subtests of the German version of the Wechsler Intelligence Scale—Fourth Edition (WAIS‐IV) were administered (Wechsler 2008). The vocabulary subtest is a verbal test that measures word knowledge and the ability to verbally express definitions of words. Matrices reasoning is a nonverbal reasoning task that requires individuals to identify patterns in designs. Raw scores are converted to age‐scaled scores.

#### 2.2.2 PM Tasks

To assess experimenter‐assigned event‐based PM, once a day, a PM cue (i.e., a word written in capital letters) was embedded in one of the ESM daily questionnaires. The participants were instructed to respond to the questionnaire with a signal word by sending a text message with the content: “word found” to the investigator. The dependent variable was the number of text messages received (= correctly fulfilled event‐based PM tasks; three in total). The participants had to respond within 30 min of receiving the message for the response to be considered a correctly solved PM task. To measure time‐based PM, participants were instructed to text the research team during a specific time period of 2 h (e.g., 12–14 PM), which was communicated to them via a separate text message each morning at 10 AM. Participants were asked not to use a reminder for this purpose. The time period during which the message had to be written varied over the test period. The dependent measure was the number of text messages received on time (= correctly fulfilled time‐based PM tasks; three in total). Participants had to respond within the given time span to be considered a correctly solved PM task.

#### 2.2.3 Diary Task

To assess real‐life PM performance, participants were asked to indicate six activities that they intended to perform across the next 3 days. Of these, three activities should be activities that represented chores (e.g., housework, arranging appointments, work‐related activities etc.). The participants in our study mentioned activities such as studying for an exam, doing the laundry, sending out job applications, scheduling a doctor's appointment by phone, or cleaning the garage. The other three activities were to be activities that the participant enjoys or that are leisure activities (e.g., practicing hobbies, meeting friends, etc.). Participants mentioned activities such asgoing to the cinema, visiting the gym, horseback riding, playing video games, or drawing. Participants were informed that the planned activities should not be routine activities (e.g., brushing teeth and traveling to work by train) and should not be activities that the participant intended to implement anyway. Participants were instructed to implement the activities at the designated time. Any form of reminder was not allowed. After 3 days, participants were asked via an online questionnaire (whereby the access link was sent to them via a separate SMS) whether they implemented each planned activity and at what time. Reasons for late or non‐implementation of the planned activities were also noted. The dependent variable was the number of activities (obligations vs. leisure activities) performed on time.

#### 2.2.4 Experience Sampling Method

Participants were instructed that over a three‐day period, they would receive six daily text messages at random times between 10 am and 6 pm containing a link to a questionnaire. The questionnaire asked participants to outline which activity they were doing at that moment. After data collection, in line with Ilen et al. (2024) the experimenter allocated each activity to one of the following *activity types*: work/school activity (e.g., writing a term paper, attending a meeting), eat/house‐related activity (e.g., cooking and cleaning the bathroom), physical activity/sports (e.g., visiting the gym and going for a walk), other leisure activity (all other leisure activities except for sports and physical activity‐related activities, e.g., reading, playing video games), social activity (e.g., meeting with a friend and watching a movie with someone), nothing/resting (e.g., lying in bed and waiting for a doctor's appointment).

It was assessed whether the participants had planned to do this specific activity at this time (dichotomous scale, yes or no). Participants were also asked to indicate on a dichotomous scale if, so far, their day had gone according to plan and if not, they were prompted to briefly outline the reasons. These variables were intended to measure participants' daily structure (= *daily structure*). Rating scales (response format five‐point Likert scale; 1 = Disagree, 5 = Strongly Agree) were used to assess personal importance (“This activity is important to me”), and importance to other people (social importance) of a specific activity (“Doing this activity is important to other people”). In addition, their intrinsic motivation was measured (“I don't need a reward for activities like this, I enjoy them so much”). All Items were taken from the Questionnaire on Current Motivation (QCM, Rheinberg et al. 2019). This resulted in three activity characteristics variables: *importance*, *importance for others*, and *motivation*. Two items of the Trait Well‐Being Inventory ‐Mood Level Scale—German short version (Dalbert 2002; “I feel exhausted and tired” and “I feel stressed and nervous”) were used (response format five‐point Likert scale; 1 = Disagree, 5 = Strongly Agree) and participants were asked to indicate the reasons for the stress experience if they experienced any. The average of the two items of the questionnaire resulted in the variable *perceived stress*. The completion time of a questionnaire was about 1–2 min. Participants were instructed to complete the questionnaire as soon as possible after receiving it. For each participant, the average of the 18 ESM activity characteristics variables (importance, importance for others, motivation and stress) was calculated to obtain a measure of the average level during the test period.

#### 2.2.5 Procedure

**FIGURE 1:**
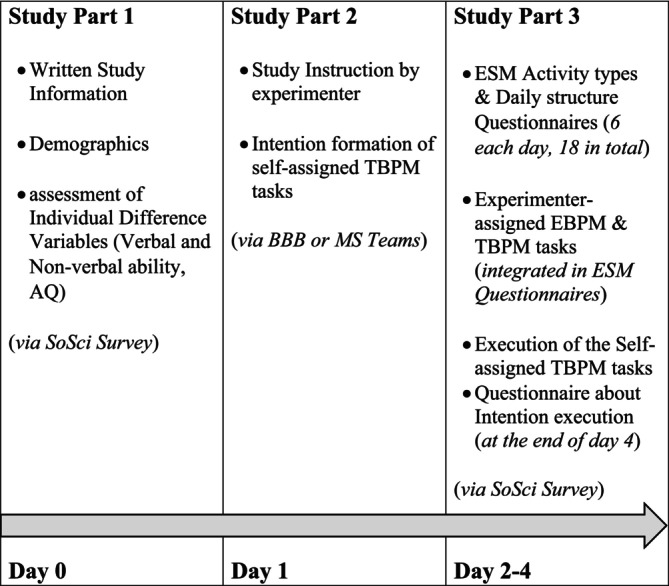
Overview of the study timeline.

An overview of the study timeline is provided in Figure 1. Before taking part in the study, participants were asked to give written informed consent. They were then requested to answer a questionnaire assessing sociodemographic information and fill in the Autism Spectrum Quotient Test—Short Version (Freitag et al. 2007). All written data was collected via the online platform SoSci Survey (Leiner 2019). Following the completion of the online questionnaire, participants could sign up for an appointment for the online testing. As part of the online testing via Microsoft Teams and/or BigBlueButton, the vocabulary and matrices reasoning subtests of the WAIS‐IV were first conducted with the participants. Introduction to this study took place via a 30‐min video conference using the platform Microsoft Teams or BigBlueButton.

#### 2.2.6 Statistical Analyses

All data was analyzed using IBM SPSS Statistics (Version 27) and JASP (JASP Team 2023, Version 0.17.3). Group comparisons for PM performance were done using one‐way and mixed ANOVAs. Additional Bayesian statistics were conducted to estimate the strength of evidence for the null hypothesis. Mediation analyses and Bayesian ANOVAs were implemented in JASP. The support for our hypotheses is described by the Bayes factor (BF). The *BF*_10_ describes the ratio between the evidence for the hypothesis *H*_1_ relative to the null hypothesis *H*_0_ (see van den Bergh et al. 2020).

Group comparisons for activity types and daily structure were examined using the chi square test.

To explore the relationships between average perceived stress and PM performances, we conducted correlation analyses in both experimental groups.

To analyze the EMA data, which have a two‐level structure, with repeated measurements (Level 1) nested within individuals (Level 2), multilevel regression models were used. For activity characteristics variables (e.g., Stress and Importance), group comparisons were done using multilevel regression models with random intercepts. To examine the associations between the importance of activities (Importance, Importance to others, and Motivation) and perceived stress, separate multilevel regression models with random intercepts and random slopes were estimated, with each importance variable, group, and their interaction as independent variables and stress as a dependent variable.

## 3 Results

### 3.1 PM Performance

The results of the group comparisons of PM performance are presented in Table 2. To compare PM performance in the experimenter‐assigned PM tasks across groups, a two‐way mixed ANOVA with group (ASD, Controls) as a between‐subjects variable and type of PM task (event‐based PM, time‐based PM) as a within‐subjects variable was conducted. The analysis revealed a significant main effect of type of PM task, *F*(1,57) = 103.05, *p* < 0.01, $\eta_{p}^{2}$ = 0.64. Bonferroni‐adjusted post hoc analyses indicated a superiority (*p* < 0.01) of time‐based compared to event‐based PM; (0.43, 95% CI [0.36, 0.52]). The main effect of group was not significant, *F* < 1. There was no significant interaction effect, *F* < 1.

**TABLE 2:** Group comparison of PM performance.

	ASD (*n* = 29)	CG (*n* = 30)	*F* (df)	*p*	*η* ^2^	BF_10_ ^a^
	*M* (SD)	*M* (SD)				
Experimenter‐assigned PM tasks						
Event‐based PM	0.34 (0.33)	0.28 (0.28)	0.68	0.413	0.012	0.352
Time‐based PM	0.70 (0.28)	0.79 (0.27)	1.49	0.226	0.147	0.494
Self‐assigned time‐based PM tasks						
Obligations	0.78 (0.24)	0.72 (0.29)	0.77	0.385	0.014	0.369
Leisure activities	0.72 (0.31)	0.78 (0.30)	0.48	0.491	0.001	0.328
Total	0.77 (0.19)	0.75 (0.23)	0.15	0.701	0.193	0.285

Abbreviations: ASD, autism spectrum disorders; CG, control group.

^a^ BF_10_ describes the likelihood of the data under H_1_ compared to H_0_, BF_10_ = 1/3–1/10, moderate evidence for H_0_; BF_10_ = 1–1/3, anecdotal evidence for H_0_.

To explore possible differences in performance in self‐assigned PM tasks, a mixed ANOVA with group (ASD, Controls) as a between‐subjects variable and type of intention (obligations, leisure activities) as a within‐subjects variable was carried out. There were no significant differences in PM performance between groups, *F* < 1, and no main effect of type of intention, *F* < 1. Again, there was also no significant interaction effect, *F* < 1.

#### 3.1.1 Correlations of PM and Perceived Stress

Correlational analyses were conducted to investigate possible relations between average perceived stress and PM performance separately for both groups (see Tables 3 and 4). In the ASD group, a higher overall score in the AQ was associated with better time‐based PM performance regarding self‐assigned obligations and worse time‐based PM performance regarding self‐assigned leisure activities. Higher non‐verbal abilities were associated with lower performance in the experimenter‐assigned time‐based PM tasks and the self‐assigned PM obligations in the ASD group. A higher average stress level in the ASD group was related to poorer performance in the self‐assigned PM leisure activities.

**TABLE 3:** Correlations of study variables in the ASD group.

	1	2	3	4	5	6	7	8	9
Individual difference variables									
(1) AQ – Total Score									
(2) WAIS.IV‐NVA	0.02								
(3) WAIS‐IV‐VA	−0.15	0.06							
(4) Stress	0.21	0.07	−0.11						
Experimenter‐assigned PM tasks									
(5) Event‐based PM	−0.05	0.09	0.24	−0.27					
(6) Time‐based PM	−0.15	−0.47*	0.09	0.05	0.41*				
Self‐assigned time‐based PM tasks									
(7) Obligations	0.54**	−0.34	−0.43*	0.29	−0.21	0.07			
(8) Leisure activities	−0.43*	−0.16	−0.17	−0.40*	0.21	0.22	−0.08		
(9) Total	−0.02	−0.29	−0.42*	−0.13	−0.08.	0.19	0.45**	0.71**	

*Note:* *p < 0.05, **p < 0.01.

**TABLE 4:** Correlations of study variables in the control group.

	1	2	3	4	5	6	7	8	9
Individual difference variables									
(1) AQ – Total score									
(2) WAIS.IV‐NVA	0.15								
(3) WAIS‐IV‐VA	−0.04	0.56**							
(4) Stress	−0.06	−0.11	0.03						
Experimenter‐assigned PM tasks									
(5) Event‐based PM	0.02	−0.03	0.10	−0.12					
(6) Time‐based PM	−0.39*	−0.18	−0.10	−0.14	0.38*				
Self‐assigned time‐based PM tasks									
(7) Obligations	−0.17	0.13	0.29	−0.05	0.22	0.54**			
(8) Leisure activities	0.30	0.24	0.21	−0.22	0.17	0.07	0.19		
(9) Total	0.08	0.23	0.31	−0.17	0.24	0.38*	0.79**	0.79**	

*Note:* *p < 0.05, **p < 0.01.

In the control group, a higher AQ total score was associated with poorer performance in the experimenter‐assigned time‐based PM tasks. Better performance in the experimenter‐assigned time‐based PM tasks was correlated with better performance in the self‐assigned time‐based obligations in the control group.

### 3.2 Experience Sampling

Participants responded to the ESM questionnaire 993 times, with 492 responses by the ASD group and 583 by the control group. There were no group differences on the average completion of ESM questionnaires, *X* ^2^ (1) = 0.03, *p* = 0.862.

#### 3.2.1 Activity Types and Daily Structure

The frequencies of activity types and daily structure of both groups and group comparisons can be found in Table 5.

**TABLE 5:** Descriptive statistics and group comparisons of activity types and planning behavior.

	ASD (*n* = 29)	CG (*n* = 30)	*χ* ^2^ (df)	*p*
	*M* (SD)	*M* (SD)		
Type of activity				
Leisure activity	136 (27.6%)	113 (22.55%)	2.31 (1)	0.142
Work/school activity	147 (29.88%)	152 (33.34%)	0.291 (1)	0.590
Eat/house‐related activity	132 (26.83%)	98 (19.56%)	5.78 (1)	0.016
Physical activity/sports	35 (7.11%)	54 (10.78%)	4.86 (1)	0.028
Social activity	31 (6.3%)	38 (7.58%)	0.910 (1)	0.204
Nothing/resting	11 (2.23%)	28 (5.59%)	7.69 (1)	0.006
Total activities	492 (100%)	483 (100%)	0.03 (1)	0.862
Daily structure				
Activities (planned/not‐planned)	308/185	343/143	7.21 (1)	0.007
Implementation (in time/not in time)	208/279	264/213	15.4 (1)	0.001
Days proceeding (as planned/not as planned)	327/165	376/110	14.38 (1)	0.001

Abbreviations: ASD, autism spectrum disorders; CG, control group.

Chi‐square tests indicated that autistic participants stated engaging in more eat/house‐related activity, *X* ^2^ (1) = 5.78, *p* = 0.016, and fewer physical activities, *X* ^2^ (1) = 4.86, *p* = 0.028, compared to control participants. Control participants were more likely than autistic participants to report engaging in no activity, *X* ^2^ (1) = 7.69, *p* = 0.006.

Autistic participants planned their daily activities significantly less often in advance compared to control participants, *X* ^2^ (1) = 7.21, *p* = 0.007, and implemented significantly fewer of these activities at the planned time compared to control participants, *X* ^2^ (1) = 15.4, *p* = 0.001. In comparison to autistic participants, control participants significantly more often stated that their day had proceeded as planned, *X* ^2^ (1) = 14.38, *p* = 0.001.

#### 3.2.2 Group Comparisons for Perceived Stress and Activity Characteristics

A multilevel analysis with perceived stress as the dependent variable showed that autistic participants reported higher levels of perceived stress in their daily lives than non‐autistic participants. The results can be found in Table 6.

**TABLE 6:** Descriptive statistics and group comparisons of perceived stress and activity characteristics.

	ASD (*n* = 29)	CG (*n* = 30)	ASD vs. CG	
	*M* (SD)	*M* (SD)	*β* (95% CI)	*p*
Stress	2.25 (1.02)	1.87 (0.80)	0.38 (0.04 to −72)	0.029
Importance	3.73 (0.52)	3.84 (0.49)	−0.12 (−0.38 to 0.14)	0.351
Importance to others	2.88 (0.68)	2.96 (0.64)	−0.09 (−0.42 to 0.24)	0.591
Motivation	3.11 (0.69)	3.30 (0.61)	−0.17 (−0.51 to 0.16)	0.318

Abbreviations: ASD, autism spectrum disorders; CG, control group.

#### 3.2.3 Effects of Activity Characteristics on Perceived Stress

Separate multilevel regression models with activity characteristics (Importance and Importance to others and Motivation) as independent variables and perceived stress as the dependent variable showed that motivation and importance of the activity were significantly negatively associated with perceived stress in both groups. All results can be found in Table 7.

**TABLE 7:** Associations of activity characteristics and activity characteristics × group interaction with perceived stress, controlling for effects of age and gender.

	*β* (95% CI)	*p*
Outcome: stress		
Age	0.01 (−0.01 to 0.03)	0.235
Age × group	−0.03 (0.05 to 0.00)	0.056
Gender	0.20 (−0.19 to 0.59)	0.302
Gender × group	−0.25 (−0.89 to 0.40)	0.448
Activity characteristics		
Importance	−0.11 (−0.18 to −0.04)	0.002
Importance × group	−0.06 (−0.03 to 0.16)	0.183
Importance to others	0.03 (−0.02 to 0.07)	0.267
Importance to others × group	−0.02 (−0.09 to 0.05)	0.589
Motivation	−0.18 (−0.23 to −0.13)	0.001
Motivation × group	0.05 (−0.02 to −0.13)	0.150

## 4 Discussion

PM difficulties in autism have been shown by several studies (Altgassen et al. 2009, 2019; Brandimonte et al. 2011; Henry et al. 2014; Kretschmer‐Trendowicz et al. 2014; Landsiedel and Williams 2020; Williams et al. 2013; Williams et al. 2014; Yi et al. 2014). However, there are also several studies demonstrating intact (event‐based) PM in autistic individuals (Altgassen et al. 2010; Altgassen and Koch 2014; Groenman et al. 2024; Henry et al. 2014; Sheppard et al. 2016; Williams et al. 2013). Importantly, previous studies exclusively used laboratory‐based PM tasks; hence it is unknown how autistic individuals perform in everyday life and if the lab‐based findings can be transferred to everyday life. The aim of the present study was therefore to assess PM abilities in autism in everyday life using both naturalistic, experimenter‐assigned (event‐based and time‐based) and self‐assigned PM tasks (categorized into leisure activities and obligations).

Contrary to our expectations, we did not find any group differences in performance between autistic and control participants in any of the various PM tasks—neither with regards to experimenter‐ nor self‐assigned PM tasks. Therefore, the results might suggest that autistic adults show no difficulties in the execution of intentions in everyday life.

The completion of naturalistic PM tasks might be easier for autistic individuals than having to work on lab‐based PM tasks. Attending assessments in the laboratory could pose additional stress for autistic participants who often have a preference for routines (APA 2013) and intolerance regarding uncertain situations (Boulter et al. 2014; South and Rodgers 2017; Wigham et al. 2015). Being in novel situations during testing may elicit stress and excitement and could affect memory performance and executive functioning, which are both involved in PM (see Shields et al. 2016).

We assumed that participants might be more motivated to engage in self‐assigned activities than in experimenter‐assigned PM tasks, leading them to potentially execute the self‐assigned activities more successfully. Self‐assigned PM tasks, as opposed to experimenter‐assigned tasks, are set by the participants themselves and are thus more likely to be intrinsically motivated (Deci and Ryan 1985; Ryan and Deci 2000). These tasks may also hold greater personal significance, potentially enhancing PM performance. The self‐assigned time‐based PM tasks in our study were divided into obligations and leisure activities. However, given that no group differences in any of the PM task conditions emerged, we cannot make a clear statement regarding the influence of motivational factors on everyday PM performance in ASD based on the results of this study. Indeed, performance in all PM tasks was very high in both groups (70%–80% completed PM tasks), except for the event‐based PM tasks assigned by the experimenter, suggesting that participants found the tasks easy or were generally motivated to perform them correctly.

Unlike typical laboratory PM paradigms, our self‐assigned PM tasks were not embedded in standardized ongoing tasks that were the same for all participants. In fact, we did not assess whether the participants were engaged in another activity at the time of the PM tasks. The absence of an allocated ongoing task may have led participants to perceive the PM tasks themselves as highly important for the experiment, potentially contributing to the good PM performance. Landsiedel and Williams (2020) demonstrated that emphasizing the importance of the PM task increased time‐based PM performance of autistic participants. Additionally, given that no standardized ongoing tasks were assigned that needed to be interrupted to perform the experimenter‐assigned PM tasks, ongoing task absorption and thus cognitive demands may have varied across participants and may have been lower compared to typical laboratory PM paradigms, which may have left participants with more cognitive resources to perform the PM tasks, resulting in increased PM performance. In the case of the self‐assigned PM tasks, being aware of the requirement to complete the PM tasks within the set time frame might have led to planning and daily structuring around the PM tasks, which could explain why autistic participants performed these tasks as successfully as those in the control group.

In contrast to previous research (Kliegel et al. 2008; McDaniel and Einstein 2007) all time‐based PM tasks were performed significantly better than event‐based PM tasks. This could be attributed to methodological limitations of the experimenter‐assigned event‐based PM task. First, participants might have forgotten the instructions to respond to the cue (bolded word) over the duration of time. In contrast, for the experimenter‐assigned time‐based tasks, the instructions were repeated in written form on the morning of the task, potentially enabling participants to perform the tasks more successfully. However, it is more likely that the cue was not sufficiently salient, leading to potential oversight.

Furthermore, ceiling effects may have contributed to our inability to find performance differences in the various PM task conditions (obligation vs. leisure activity, self‐assigned vs. experimenter‐assigned) and could also explain why we did not find group differences between the ASD and control groups. One could also question to what extent our self‐assigned PM tasks were truly self‐assigned given that, ultimately, participants were still instructed to perform specific activities within the framework of a scientific experiment, which may have pressured them to ensure the tasks were carried out. Therefore, it is not entirely clear whether the self‐assigned PM tasks differed from experimenter‐assigned PM tasks in their perceived autonomy and thus their intrinsic motivational value.

Finally, it should be noted that we did not conduct a PM laboratory task, which would have allowed us to directly compare PM performance in the lab and in a naturalistic setting within the same sample. This makes it more difficult to draw valid conclusions about potential setting‐dependent PM differences in autism. Future studies should integrate both laboratory and naturalistic PM tasks into the study design to enable a comparison between lab‐ and everyday PM performance within the same sample.

The experimenter‐assigned PM tasks were integrated into a three‐day ESM task, during which we assessed the types of activities participants engaged in, their motivation to perform them, and their perceived stress levels. We hoped to gather additional information about participants' activities and daily structure, especially in light of the assigned PM tasks performed during the same time period.

In contrast to acute stress, chronic stress is believed to significantly impair PM performance (Chen et al. 2019; Eskildsen et al. 2015; Schnitzspahn et al. 2011; Stewart and McFarland 2020). Specifically, autistic individuals are thought to be particularly vulnerable to experiencing chronic stress in their daily lives (Bishop‐Fitzpatrick et al. 2015, 2017; Hirvikoski and Blomqvist 2015; McGillivray and Evert 2018; Moseley et al. 2021) which may negatively affect their PM performance. Interestingly, correlational analyses only revealed a significant association between self‐assigned time‐based leisure PM tasks and average perceived stress in the ASD group, indicating that lower PM performance of self‐assigned leisure activities was related to higher stress levels. This could suggest that when feeling stressed, participants tend to skip the self‐assigned leisure activities but not the obligations. However, this remains purely a speculative assumption. Differences in mean perceived stress between self‐assigned PM tasks and experienced stress during other activities could not be confirmed for either group, possibly due to the small sample size.

The types of ESM activities performed did not differ between the ASD and control groups, except for household‐related and physical activities. Interestingly, ASD participants reported engaging in social activities at a similar frequency as control participants, contrary to what might be expected given the often reported altered social motivation (Dichter 2018; Clements et al. 2018) or increased prevalence of social anxiety and its associated avoidance behavior in ASD (e.g., Kuusikko‐Gauffin et al. 2013; Spain et al. 2018). It is worth noting that similar to control participants, many autistic participants reported interactions with their spouse, which may have contributed to the higher frequency of reported social interactions. Additionally, our sample included a higher proportion of women than typically found in ASD studies. There is evidence suggesting that the social motivation of autistic women does not differ from that of non‐autistic women (Sedgewick et al. 2016) which could also explain why we did not find differences in the frequency of social activities in our study.

Autistic participants also appeared to plan their ESM activities less frequently in advance compared to participants in the control group. Considering the high need for predictability and routines in ASD, these results may seem surprising at first. One possible explanation could be that the autistic participants intentionally planned fewer activities in advance to avoid potential deviations from the plan and to avoid the associated stress and uncertainty. At this point, this explanation remains purely speculative and needs to be investigated by future studies.

Similarly, autistic participants more often reported that planned activities were not carried out as scheduled and their day did not go as planned. These results align with previous research findings (Happé et al. 2006; Hill 2004; van den Bergh et al. 2014) that have suggested planning difficulties in autistic individuals. Interestingly, our PM tasks, which also required planning, were successfully implemented by autistic participants. A reason for this could be that the planning of the PM tasks was discussed with the participants during the study instructions, which may have facilitated the timely execution of these activities. This would imply that encoding support in the intention formation phase (Kliegel et al. 2002) could lead to improved PM performance in autism. Another possible reason could be that motivation or the perceived social expectation stemming from study participation encouraged participants to prioritize the completion of the planned activities within the study. This would suggest that motivational factors may also play a role in successful PM performance.

Finally, we did not find any group differences with regard to subjective importance of activities and subjective motivation to perform the activities. This suggests that autistic participants in our study were as motivated to carry out the activities and rated the activities as important as participants in the control group. However, consistent with previous ESM studies (Ilen et al. 2023; van der Linden et al. 2021), autistic participants rated their stress levels on average higher than participants in the control group did. Across both groups, lower perceived personal importance of the activity and lower motivation to carry out the activity were related to higher levels of perceived stress. These results suggest that autistic individuals, like non‐autistic individuals, may feel more stressed when they have to engage in activities that are less meaningful to them and for which they are less motivated.

In summary, we found no performance differences between autistic and non‐autistic participants in our naturalistic PM tasks. Indeed, congruent with previous studies (Bishop‐Fitzpatrick et al. 2015, 2017; Hirvikoski and Blomqvist 2015; McGillivray and Evert 2018; Moseley et al. 2021; ESM studies: Ilen et al. 2023; van der Linden et al. 2021), autistic participants experienced more stress during the test period than controls. However, we did not find any associations between PM performance and perceived stress. Lower perceived personal importance of ESM activities as well as lower motivation were related to a higher perceived level of stress in autistic and non‐autistic participants. Future studies should investigate whether factors stemming from the laboratory condition rather than the actual PM task could affect the PM performance of autistic individuals. At the same time, attention should be paid to identifying factors that could enhance the PM performance of autistic individuals in naturalistic settings. Although based on our study results, we cannot make clear statements about how motivation could affect the execution of PM tasks in autistic individuals, it remains debatable whether motivational factors are crucial in the performance of autistic individuals, both in PM and other experimental tasks, and play a role in everyday behavior.

## Conflicts of Interest

The authors declare no conflicts of interest.

## Supporting information


**Appendix S1.** Supplementary Information.
**Table S1.** Mean and standard deviations of activity characteristics of self‐assigned PM tasks and not PM related activities in the ASD group and the control group.
**Table A2.** Comparison of activity characteristics of self‐assigned PM tasks and not PM related activities. Effects of activity type, group and interaction between activity type and group.

## Data Availability

The data that support the findings of this study are available from the corresponding author upon reasonable request.
