# Supplementary material for: Prospective Memory Performance of Autistic Adults in Everyday Life: The Role of Stress and Motivation
Source: Autism Res. 2025 May 29;18(7):1447–60. doi: 10.1002/aur.70057 (PMC12279003; doi:10.1002/aur.70057)
Supplement: Supplementary file 1 — Appendix S1. Supplementary Information. Table S1. Mean and standard deviations of activity characteristics of self‐assigned PM tasks and not PM related activities in the ASD group and the control group. Table A2. Comparison of activity characteristics of self‐assigned PM tasks and not PM related activities. Effects of activity type, group and interaction between activity type and group. [file AUR-18-1447-s001.pdf]

Appendix

Table A1. Mean and standard deviations of activity characteristics of self-assigned PM tasks and not PM related activities in the ASD group and the control group

	**Importance**		**Importance to Others**		**Motivation**		**Stress**	
	**ASD Group (n=16)**	**Control Group (n=18)**	**ASD Group (n=15)**	**Control Group (n=18)**	**ASD Group (n=16)**	**Control Group (n=18)**	**ASD Group (n=16)**	**Control Group (n=19)**
	***M*(SD)**	***M*(SD)**	***M*(SD)**	***M*(SD)**	***M*(SD)**	***M*(SD)**	***M*(SD)**	***M*(SD)**
Self-assigned PM tasks	3.62 (.84)	4.09 (.80)	2.39 (1.19)	2.67 (.93)	2.93 (1.27)	3.51 (1.31)	2.65 (.80)	1.93 (.84)
Not PM related activities	3.58 (.55)	3.84 (.49)	2.84 (.72)	3.05 (.73)	2.53 (.69)	3.14 (.54)	2.50 (.80)	1.95 (.55)

Note. ASD=autism spectrum disorders group; CG=control group. Only participants who had mentioned self-assigned PM tasks as part of their reported ESM activities were included in the analysis. Average scores per person were calculated for the activity characteristics of the self-assigned PM tasks and for the activity characteristics of the remaining ESM activities (= *Not PM related activities*).

Table A2. Comparison of activity characteristics of self-assigned PM tasks and not PM related activities. Effects of activity type, group and interaction between activity type and group

	**Activity Type (PM/ Not-PM)**			**Group (ASD/Control)**			**Interaction**		
	***F*(df)**	***p***	***η²***	***F*(df)**	***p***	***η²***	***F*(df)**	***p***	***η²***
Importance	1.17 (32)	.287	.035	3.41 (32)	.074	.01	.611 (32)	.449	.019
Importance to others	3.91 (31)	.057	.112	1.00 (31)	.325	.031	.031 (31)	.862	.001
Motivation	3.31 (32)	.079	.096	5.23 (32)	.029	.144	.003 (32)	.955	.000
Stress	1.79 (33)	.191	.053	5.77 (33)	.022	.153	2.59 (33)	.117	.075

Note. A mixed ANOVA (Group/Activity Type) was conducted. Only participants who had mentioned self-assigned PM tasks as part of their reported ESM activities were included in the analysis. Average scores per person were calculated for the activity characteristics of the self-assigned PM tasks and for the activity characteristics of the remaining ESM activities (= *Not PM related activities*).
